# Maternal Whole Blood Gene Expression at 18 and 28 Weeks of Gestation Associated with Spontaneous Preterm Birth in Asymptomatic Women

**DOI:** 10.1371/journal.pone.0155191

**Published:** 2016-06-22

**Authors:** Yujing J. Heng, Craig E. Pennell, Sheila W. McDonald, Angela E. Vinturache, Jingxiong Xu, Mary W. F. Lee, Laurent Briollais, Andrew W. Lyon, Donna M. Slater, Alan D. Bocking, Lawrence de Koning, David M. Olson, Siobhan M. Dolan, Suzanne C. Tough, Stephen J. Lye

**Affiliations:** 1 Departments of Obstetrics & Gynaecology and Physiology, University of Toronto, Lunenfeld-Tanenbaum Research Institute, Mount Sinai Hospital, Toronto, ON, Canada; 2 Department of Pathology, Harvard Medical School, Beth Israel Deaconess Medical Center, Boston, MA, United States of America; 3 School of Women's and Infants' Health, The University of Western Australia, Crawley, WA, Australia; 4 Department of Paediatrics, Cumming School of Medicine, University of Calgary, Calgary, AB, Canada; 5 Departments of Obstetrics & Gynaecology and Physiology & Pharmacology, Cumming School of Medicine, University of Calgary, AB, Canada; 6 Dalla Lana School of Public Health, University of Toronto, ON, Canada; 7 Department of Pathology and Lab Medicine, University of Saskatchewan, St Paul’s Hospital, Saskatoon Health Region, Saskatoon, SK, Canada; 8 Calgary Laboratory Services, Calgary, AB, Canada; 9 Department of Community Health Sciences, Cumming School of Medicine, University of Calgary, Calgary, AB, Canada; 10 Departments of Obstetrics & Gynecology, Physiology and Pediatrics, University of Alberta, Edmonton, AB, Canada; 11 Department of Obstetrics & Gynecology and Women's Health (Reproductive Genetics), Albert Einstein College of Medicine, Bronx, NY, United States of America; University of Rochester, UNITED STATES

## Abstract

The heterogeneity of spontaneous preterm birth (SPTB) requires an interdisciplinary approach to determine potential predictive risk factors of early delivery. The aim of this study was to investigate maternal whole blood gene expression profiles associated with spontaneous preterm birth (SPTB, <37 weeks) in asymptomatic pregnant women. The study population was a matched subgroup of women (51 SPTBs, 114 term delivery controls) who participated in the All Our Babies community based cohort in Calgary (n = 1878). Maternal blood at 17–23 (sampling time point 1, T_1_) and 27–33 weeks of gestation (T_2_) were collected. Total RNA was extracted and microarray was performed on 326 samples (165 women). Univariate analyses determined significant clinical factors and differential gene expression associated with SPTB. Thirteen genes were validated using qRT-PCR. Three multivariate logistic models were constructed to identify gene expression at T_1_ (Model A), T_2_ (Model B), and gene expression fold change from T_1_ to T_2_ (Model C) associated with SPTB. All models were adjusted for clinical factors. Model C can predict SPTB with 65% sensitivity and 88% specificity in asymptomatic women after adjusting for history of abortion and anaemia (occurring before T_2_). Clinical data enhanced the sensitivity of the Models to predict SPTB. In conclusion, clinical factors and whole blood gene expression are associated with SPTB in asymptomatic women. An effective screening tool for SPTB during pregnancy would enable targeted preventive approaches and personalised antenatal care.

## Introduction

Preterm birth (PTB; birth before 37 weeks of gestation) is the greatest challenge facing contemporary obstetrics in both high and low resource settings. The World Health Organization estimated that 11% of all live births in 2010 were premature (15 million) and PTB rates are increasing [1]. Preterm related complications include death, lifelong sequelae including motor and sensory impairment [2] and immediate and long-term emotional and financial consequences for families, communities and the health care system [3, 4]. The prevention of PTB is essential for accelerating progress towards the United Nation’s Fourth Millennium Development Goal as the social and economic benefits of reducing the rate of PTB are enormous [5].

PTB is becoming a preventable disease. The use of progesterone [6–8], cervical cerclage [9] and antibiotics [10] in women at high risk of PTB are improving outcomes. However, these treatments are only useful in a subset of women [6–8]. The current screening tools to identify asymptomatic women at high risk of spontaneous PTB (SPTB) include clinical risk factor assessment [11], measuring cervical length [12, 13] and screening for fetal fibronectin (fFN) [14, 15]. These tools are limited by their low sensitivities (<50%), with some as low as 8% [11, 16]. The cornerstone of preventing PTB is to reliably identify these women and develop tools for risk stratification. This will assist the development and implementation of preventive measures as well as efforts to improve the clinical management of PTB. The multifactorial aetiologies and serious consequences of PTB highlight the need for a multidisciplinary approach to identify factors predictive of PTB [17].

**Fig 1:**
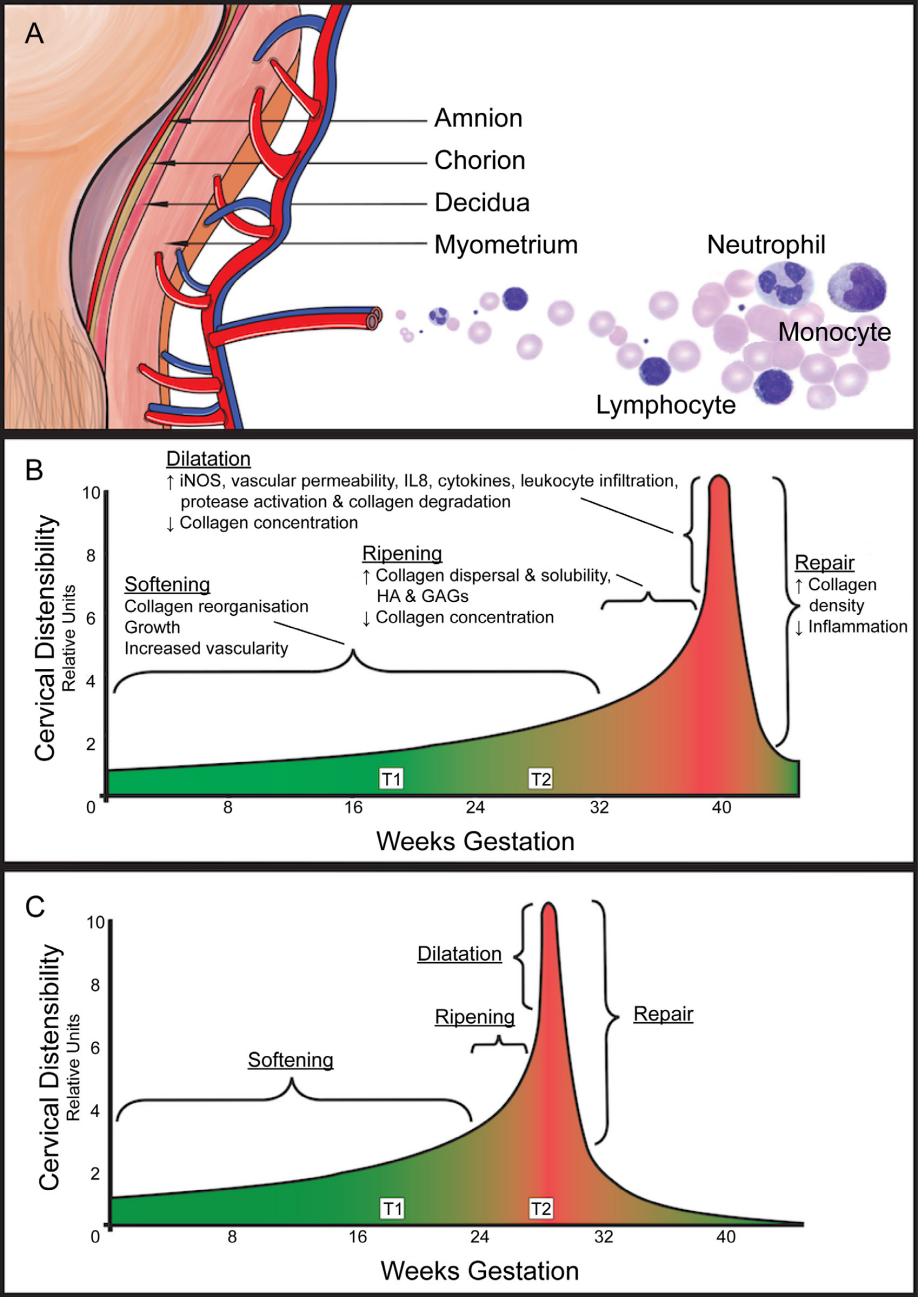
Parturition begins weeks before labour onset. **(A)** Labour is an inflammatory process with elevated levels of maternal circulating leukocytes and increased leukocyte infiltration into the myometrium, decidua and cervix before and during labour. **(B)** Parturition is a long complex process that begins weeks before the onset of labour. The cervix gradually ripens and the myometrium switches from a quiescent to a contractile state. **(C)** In preterm birth, the cascade of events culminating in birth is transposed earlier in gestation. iNOS, induced nitric oxide synthase; IL8, interleukin 8; HA, hyaluronan; GAGs, glycosaminoglycans; T1, study samples collected at 17–23 weeks of gestation; T2, study samples collected at 27–33 weeks of gestation. Illustrations adapted from Word *et al* [18].

Parturition is a complex process that begins weeks before labour onset (Fig 1) [18, 19]. Labour is an inflammatory process with elevated levels of maternal circulating leukocytes [20] and increased leukocyte infiltration into the myometrium, decidua and cervix before and during labour [21, 22]. Whole blood mRNAs are assumed to be contributed, in part, by leukocytes and may reflect physiological processes. We postulate that maternal leukocytes circulating through gestational tissues (i.e. amnion, chorion, decidua, myometrium and cervix) during pregnancy are exposed to ‘signals’ from these tissues, and respond by altering their gene expression. An alternate postulate is that maternal leukocytes may be initiating and coordinating the process of parturition. This current study profiled whole blood mRNA collected from asymptomatic pregnant women and investigated the univariate association of whole blood gene expression at approximately 18 and 28 weeks of gestation with impending SPTB. Three multivariate models associated with SPTB were subsequently constructed using data at 18 weeks, 28 weeks, and from 18 to 28 weeks of gestation.

## Materials and Methods

### Patient Recruitment

The study population was drawn from a subset of women who participated in the All Our Babies (AOB) study, a community based longitudinal pregnancy cohort in Calgary, Alberta, Canada approved by the Conjoint Health Research Ethics Board, University of Calgary (Ethics #20821 and #22128). Pregnant women receiving prenatal viral serology testing were recruited through a partnership with Calgary Laboratory Service between May 2008 and December 2010. Written consent was obtained at the time of the first blood collection. Women also completed a survey about lifestyle, psychosocial and health care utilisation (prenatal care, social support, symptoms of stress, anxiety and depression, and breastfeeding) at <25 weeks, 34–36 weeks of gestation and 4 months postpartum [23].

**Fig 2:**
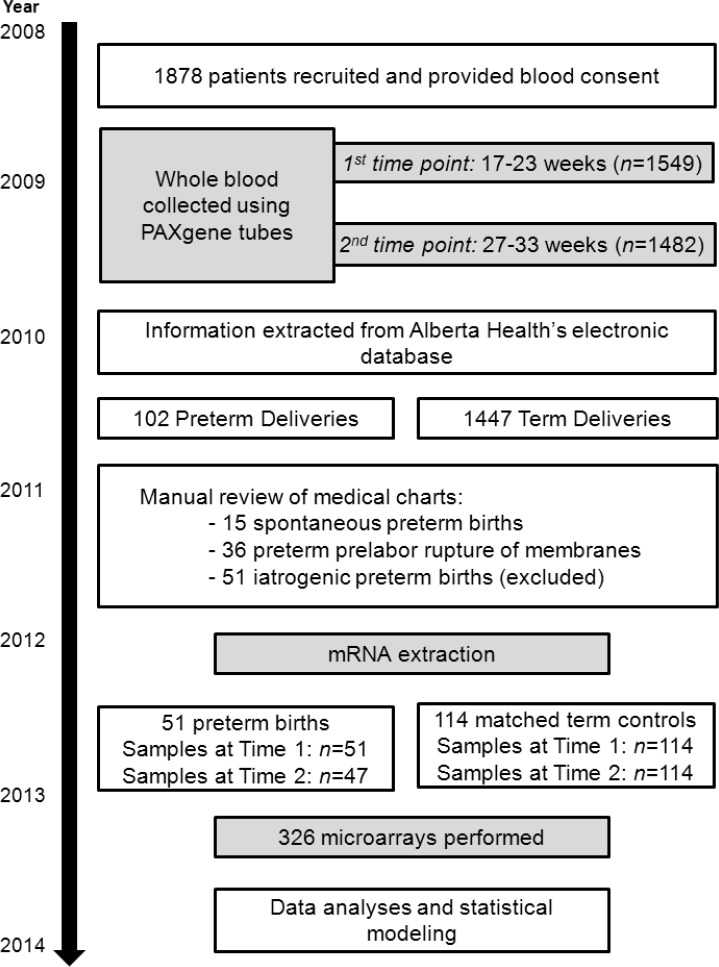
Flowchart outlining the recruitment, patient phenotyping and sample selection process for this study.

Detailed inclusion and exclusion criteria for the AOB study have been described [24]. Briefly, inclusion criteria were ≥18 years of age, gestation age <18 weeks at time of recruitment and singleton pregnancy. Exclusion criteria were multifetal pregnancy and pre-existing medical conditions (diabetes, high blood pressure, autoimmune disorders, kidney disease, cardiovascular disease or chronic infection). Clinical and antenatal records were extracted from the Alberta Health electronic database. Women who had PTB were confirmed by a manual review of the medical charts. Clinical data were unavailable for four women who delivered out of province at term. Fig 2 summarises the patient recruitment, patient phenotyping and selection process.

Spontaneous preterm labour (SPTL) is defined as spontaneous onset of labour ≤37 weeks of gestation resulting in preterm delivery. Preterm prelabour rupture of membranes (PPROM) is defined as spontaneous rupture of membranes at <37 weeks without labour, onset of spontaneous labour occurred at least 60 min after PPROM and subsequent preterm delivery. Term delivery is birth at ≥37 weeks of gestation irrespective of spontaneous onset or induction, vaginal delivery or caesarean section. In Calgary, anaemia is defined as <120 g/L of haemoglobin; oligohydramnios and polyhydramnios are diagnosed using an amniotic fluid index of <5 cm and >20 cm, respectively. Antepartum haemorrhage is defined as recurrent haemorrhage at ≤20 or >20 weeks of gestation. Urinary tract infection (UTI) was indicated positive by either microscopic or macroscopic urinalysis, or culture. Demographic, clinical, labour and delivery variables were analysed using one-way ANOVA, Student’s *t*-test, Chi-squared test or Fisher’s exact test (R, version 3.2.1).

### Sample Collection and Processing

Maternal blood samples were collected at 17–23 (time point 1, T_1_) and 27–33 weeks of gestation (time point 2, T_2_) into four PAXgene blood RNA tubes (PreAnalytix/BD Canada, Mississauga, ON, Canada) and stored at -80°C until analysis.

### RNA Extraction, Quality Check and Microarray

Total RNA was extracted using the PAXgene blood RNA Kit (PreAnalytix/QIAGEN, Toronto, ON, Canada) adhering to the manufacturer’s protocol. All samples had RNA integrity number of >7 (RNA 6000 Nano Kit and Agilent 2100 BioAnalyzer; Agilent Technologies, Santa Clara, CA) and were hybridised to Affymetrix Human Gene 2.1 ST (Affymetrix, Santa Clara, CA). Microarray was performed by The Centre for Applied Genomics (TCAG; The Hospital for Sick Children, Toronto, ON, Canada). Data were deposited into the National Center for Biotechnology Information Gene Expression Omnibus (accession number: GSE59491; https://www.ncbi.nlm.nih.gov/geo/query/acc.cgi?acc=GSE59491).

### Differential Gene Expression Analyses

Microarray CEL files were normalised using Robust Multi-array Average (Bioconductor, R) [25], probes were annotated using Custom (Gene)Chip Definition Files for Entrez Gene (version 18) [26], gene expression lower than the 25^th^ percentile were removed, and differential gene expression was analysed using *limma* [27] with multiple hypothesis testing (false discovery rate, FDR). *limma* analyses were adjusted for gestational age at sampling, significant demographic or clinical variables when appropriate (see S1 Text). Differential gene expression was initially performed between SPTL and PPROM at T_1_ or T_2_ to determine if any gene was differentially expressed between these two subtypes of SPTB. There was no differentially expressed gene between SPTL and PPROM, thus, SPTL and PPROM were combined into a SPTB group for all subsequent analyses. Five *limma* analyses were conducted. The first two *limma* analyses determined genes differentially expressed between women who had SPTBs and term deliveries at (1) T_1_ or (2) T_2_.

Investigating the temporal gene expression from T_1_ to T_2_ provides information about the progression of pregnancies that result in normal term deliveries or SPTBs. Hence, the third and fourth *limma* analyses were performed to identify genes displaying temporal changes from T_1_ to T_2_ within women who had (3) SPTB or (4) term deliveries. The fifth analysis was conducted to identify (5) genes whose expression fold change from T_1_ to T_2_ were different between SPTBs and term deliveries. Genes with FDR<0.05 were selected for qRT-PCR validation.

### Gene Set Enrichment

Pre-ranked Gene Set Enrichment Analyses [28] was utilised to determine significantly enriched gene sets/pathways (Gene Ontology Biological Processes, Reactome, KEGG and BioCarta, versions 5.1) between women who had SPTBs and term deliveries at (1) T_1_ or (2) T_2_; gene sets associated with temporal changes within women who had (3) SPTBs or (4) term deliveries, and (5) gene sets that reflect the difference in gene expression fold change between SPTB and term delivery.

### Qualitative Real Time PCR

Genes (*limma* FDR<0.05) that displayed >25% increase or >15% decrease, and CEL files with arbitrary intensity expression values of at least four were selected for qRT-PCR validation [29–31]. Primers were designed using Primer BLAST; pooled cDNA (paired samples from six women) were used to determine primer specificity and efficiency; and primer efficiencies (90%-105%) were determined using five-point standard curves. qRT-PCR was carried out in quadruplicate and quantification cycle (Cq) of all genes were <32. Gene expression was analysed using the 2(-Delta Delta Ct) method. Using CFX Manager 3.1 (BIO-RAD, Hercules, CA), qRT-PCR expression data were corrected for primer efficiencies and normalised to the geometric mean Cq of three optimised housekeeping genes (*TBP*, *SDHA* and *YWHAZ* [31]; average expression stability was M<0.5 [32]) to obtain the first Delta Ct. Wilcoxon test was used to compare the relative gene expression between paired samples (second Delta Ct). Correlation between microarray and qRT-PCR was performed using Spearman’s *rho*.

### Multivariate Models Associated with Spontaneous Preterm Birth

Three multivariate models were constructed to identify gene expression at T_1_ (Model A), T_2_ (Model B), and gene expression fold change from T_1_ to T_2_ (Model C) associated with SPTB (Statistical Analysis System, version 9.3, SAS Institute Inc, Cary, NC). Clinical factors occurring before T_1_ or T_2_ that were significant in univariate analyses were entered into separate clinical factor multivariate logistic regression analyses. Clinical factors occurring before T_1_ that remained significant in the multivariate clinical factor analysis were adjusted for in Model A; and significant clinical factors occurring before T_2_ in the multivariate clinical factor analysis were included for Models B and C. Gestational age were also accounted for in the Models (S1 Text). To assess validity, each Model was subjected to ten five-fold cross-validation with gene selection occurring at every fold. To evaluate the importance and effect of adjusting gene expression with clinical factors, models were also built without clinical factors (i.e. using gene expression only; S1 Text). The probability cut-off was 0.5, predictive performances such as area under receiver operator characteristic curve (ROC AUC) are the average of ten cross-validation runs. ROC AUCs were graphed using ROCR, R [33].

## Results

After excluding iatrogenic PTB, there were 51 SPTB cases where 10 were extreme SPTB (<32 weeks) and four delivered before T_2_. The average time from PPROM until labour onset was 27.7 hours. Power calculations indicated that a control group of at least 85 term women was required to match 51 SPTB, with an effect size of 0.5, significance level of 0.05 and power of 0.8. Term delivery controls (*n* = 114, power = 0.84) were matched to SPTB cases drawn from baseline survey at <25 weeks of gestation by parity (no previous birth/at least one previous birth), maternal age (<35 years versus ≥35 years), pre-pregnancy body mass index (<18.5 kg/m^2^, 18.5–24.9 kg/m^2^, 25–29.9 kg/m^2^, ≥30 kg/m^2^), ethnicity (Caucasian versus non-Caucasian), and pre-pregnancy smoking status (yes/no). A total of 326 microarrays (165 women) were performed. Eleven clinical variables were significantly associated with SPTB (Table 1).

**Table 1:** Demographic, clinical, labour and delivery characteristics of the 165 participants.

	Spontaneous Preterm Birth (SPTB)			Term Birth	SPTL vs PPROM vs Term	SPTB vs Term
	SPTL	PPROM	SPTL and PPROM		*p*-value	*p*-value
**Patient Demographics**						
Women, *n*	15	36	51	114		
Maternal age, mean years±SD	31.1±4.9	31.3±4.6	31.2±4.7	31.1±4.7	0.910	0.850
Pre-pregnancy BMI, mean±SD	21.9±2.8	26.6±9.1	25.3±8.0	25.8±72	0.321	0.702
Ethnicity					0.559	0.946
Caucasian, *n* (%)	10	29	39 (76.5)	85 (74.6)		
Non-Caucasian, *n* (%)	5	7	12 (23.5)	29 (25.4)		
Smoking during pregnancy					0.379	0.367
Yes, *n* (%)	2	8	10 (19.6)	14 (12.7)		
No, *n* (%)	13	28	41 (80.4)	96 (87.3)		
Consumption of alcohol during pregnancy					0.021	0.038
Yes, *n* (%)	3	4	7 (13.7)	4 (3.6)		
No, *n* (%)	12	32	44 (86.3)	106 (96.4)		
**Clinical Characteristics**						
Gravidity, mean±SD	2.7±1.7	2.0±1.3	2.2±1.4	2.0±1.2	0.109	0.410
Parity					0.480	0.984
Nulliparous, *n* (%)	6	21	27 (52.9)	60 (54.5)		
Multiparous, *n* (%)	9	15	24 (47.1)	50 (45.5)		
History of previous PTB					0.001	0.001
Previous PTB, *n* (%)	4	7	11 (21.6)	4 (3.6)		
No previous PTB, *n* (%)	11	29	40 (78.4)	106 (96.4)		
History of abortion					0.002	0.001
At least one abortion, *n* (%)	5	9	14 (27.5)	8 (7.3)		
No previous abortion, *n* (%)	10	27	37 (72.5)	102 (92.7)		
Mode of conception					0.188	0.267
Spontaneous conception, *n* (%)	13	34	47 (92.2)	106 (96.4)		
Assisted reproductive technologies, *n* (%)	2	2	4 (7.8)	4 (3.6)		
Oligohydramnios					0.800	1.00
Present, *n* (%)	0	2	2 (3.9)	4 (3.6)		
Absent, *n* (%)	15	34	49 (96.1)	106 (96.4)		
Polyhydramnios					0.002	0.094
Present, *n* (%)	3	0	3 (5.9)	1 (0.9)		
Absent, *n* (%)	12	36	48 (94.1)	109 (99.1)		
Gestational diabetes during pregnancy					0.216	0.350
Present, *n* (%)	2	3	5 (9.8)	5 (4.5)		
Absent, *n* (%)	13	33	46 (90.2)	105 (95.5)		
Antepartum haemorrhage during pregnancy					0.004	0.009
≥1 episode of bleeding, *n* (%)	7	9	16 (31.4)	14 (12.7)		
None, *n* (%)	8	27	35 (68.6)	96 (87.3)		
Antepartum haemorrhage <20 weeks of gestation (i.e. threatened miscarriage)					0.419	0.353
≥1 episode, *n* (%)	3	6	9 (17.6)	12 (10.9)		
None, *n* (%)	12	30	42 (82.4)	98 (89.1)		
Antepartum haemorrhage >20 weeks of gestation					0.021	0.262
≥1 episode, *n* (%)	6	4	10 (21.3)	14 (12.7)		
None, *n* (%)	8	29	37 (78.7)	96 (87.3)		
Urinary tract infection during pregnancy					<0.001	0.001
Present, *n* (%)	4	3	7 (14.0)	1 (0.9)		
Absent, *n* (%)	11	32	43 (86.0)	109 (99.1)		
Urinary tract infection before first study sample					0.029	0.029
Present, *n* (%)	1	2	3 (6.0)	0 (0.0)		
Absent, *n* (%)	14	33	47 (94.0)	110 (100.0)		
Urinary tract infection before second study sample					<0.001	0.003
Present, *n* (%)	3	2	5 (10.0)	0 (0.0)		
Absent, *n* (%)	12	33	45 (90.0)	110 (100.0)		
Anaemia during pregnancy					<0.001	<0.001
Anaemic, *n* (%)	4	8	12 (23.5)	3 (2.7)		
Non-anaemic, *n* (%)	11	28	39 (76.5)	107 (97.3)		
Anaemia before first study sample					0.099	0.099
Present, *n* (%)	0	2	2 (3.9)	0 (0.0)		
Absent, *n* (%)	15	34	49 (96.1)	110 (100.0)		
Anaemia before second study sample					<0.001	<0.001
Present, *n* (%)	4	8	12 (23.5)	1 (0.9)		
Absent, *n* (%)	11	28	39 (76.5)	109 (99.1)		
Group B Streptococcus in vaginal tract (>36 weeks of gestation)					0.071	0.043
Present, *n* (%)	2	2	4 (7.8)	24 (21.8)		
Absent, *n* (%)	13	34	47 (92.2)	86 (78.2)		
Placenta Praevia					0.143	0.327
Present, *n* (%)	0	3	3 (5.9)	2 (1.8)		
Absent, *n* (%)	15	33	48 (94.1)	108 (98.2)		
**Labour and Delivery Characteristics**						
Abruptio Placentae					0.004	0.004
Yes, *n* (%)	1	5	6 (11.8)	1 (0.9)		
No, *n* (%)	14	31	45 (88.2)	109 (99.1)		
Chorioamnionitis					0.004	0.004
Yes, *n* (%)	1	5	6 (11.8)	1 (0.9)		
No, *n* (%)	14	31	45 (88.2)	109 (99.1)		
Gestational age at delivery, mean weeks±SD	33.5±2.6	33.6±2.6	33.6±2.6	39.2±1.2	<0.001	<0.001
Birth weight, mean grams±SD	2257±551	2363±618	2332±596	3384±473	<0.001	<0.001
Neonatal Gender					0.683	0.601
Male, *n* (%)	8	22	30 (58.8)	71 (64.5)		
Female, *n* (%)	7	14	21 (41.2)	39 (35.5)		

Spontaneous preterm labour (SPTL); preterm prelabour rupture of membranes (PPROM); for continuous variables, one-way ANOVA or Student’s *t*-test was used for comparison; for categorical variables, Chi-squared test or Fisher’s test (when category size ≤4) was used.

### Differential Gene Analysis using *limma*

There was no differentially expressed gene at FDR<0.05 but at FDR<0.10, there were 0 and 26 differentially expressed genes between women who had SPTB and term delivery at T_1_ and T_2_, respectively. There were 234 and 2329 genes that displayed significant temporal differences within women who had SPTBs or term deliveries, respectively (FDR<0.05). There was no gene whose expression fold change was significantly different between SPTB and term delivery. All differential gene expression data are in S1 Table.

### Gene Set Enrichment

Significantly enriched gene sets are in S2 Table (FDR <0.05). At both sampling time points, gene sets and pathways associated with inflammation were upregulated in women with SPTBs compared to women who had term deliveries (*n* = 37 upregulated gene sets at T_1_, *n* = 103 at T_2_; 22 common gene sets). These inflammatory pathways include leukocyte migration, lysosomes, NF-kB activation, pathways involving cytokines and their receptors (e.g. IL1, IL2, IL6, IFN, IL1R, TNFR2, CCR3, CXCR4 and CD40) as well as toll-like and NOD-like receptor signalling. In contrast, women with SPTBs had lower RNA metabolism, RNA processing and T cell activation (including CTLA4 pathway) compared to women who had term deliveries (*n* = 163 downregulated gene sets at T_1_, *n* = 100 at T_2_; 77 common gene sets).

As pregnancy progressed from T_1_ to T_2_, women who had SPTBs demonstrated increased cellular proliferation, cell migration signalling pathway (by L1) and extracellular matrix degradation involving lysosomes (*n* = 32 upregulated gene sets), and decreased cellular transcription (*n* = 1 downregulated gene set). In women with term deliveries, there was increased signalling for cell migration, haemostasis, apoptosis and immune response (*n* = 114 upregulated gene sets); while there was decreased lymphocyte activation and NCAM cell adhesive interactions as pregnancy progressed to T_2_ (*n* = 36 downregulated gene sets). When investigating whether any gene set was enriched for genes whose expression fold change were different between SPTBs and term deliveries, there was no up-regulated gene set but “membrane fusion” (*n* = 1) was significantly down-regulated in SPTB.

### qRT-PCR Validation

Validation was performed on 192 samples randomly chosen from 48 women who had term deliveries (96 paired-samples) and 50 SPTBs (92 paired-samples, 4 single samples at T_1_). This resulted in using two 384-well plates to screen for each gene of interest. Genes which had significant temporal expression within women who had SPTBs or term deliveries were subjected to validation (S3 Table). Thirteen unique genes were successfully validated using qRT-PCR (*p*<0.05, Wilcoxon test). There was a significant correlation between microarray and qRT-PCR data (Spearman’s *rho* = 0.934, *p*<0.001).

### Multivariate Models Associated with Spontaneous Preterm Birth

#### Clinical Factors

Significant clinical variables determined after delivery (placental abruption, chorioamnionitis, gestational age at delivery and birth weight), during late gestation (Group B streptococcus) or those that did not achieve significance before T_2_ were not considered. Significant clinical factors with events occurring before T_1_ were alcohol consumption, history of PTB, history of abortion and UTI before T_1_. History of PTB (*p* = 0.0024) and history of abortion (*p* = 0.0025) remained significant in the clinical factor multivariate analysis and were adjusted for in Model A. Alcohol consumption, history of PTB, history of abortion, UTI before T_2_ and anaemia before T_2_ were significant clinical factors with events occurring before T_2_; history of abortion (*p* = 0.0002) and anaemia before T_2_ (*p* = 0.0003) remained significant in the clinical factor multivariate analysis and were included in Models B and C.

#### Multivariate Gene Expression Models

After adjusting for gestational age and clinical factors, candidate genes were incorporated into multivariate logistic regressions (stepwise selection) to build Models A, B and C (Table 2). As the prevalence of SPTB in this study was 31% (51 SPTB out of 165 total deliveries; higher than the average PTB rate of 10%), positive and negative predictive values, and false positive and negative rates must be interpreted with caution as these values are dependent on the prevalence of the disease, i.e. PTB in the study population, whilst sensitivity, specificity and ROC AUC are prevalence independent.

**Table 2:** Multivariate models (Models A, B and C) associated with spontaneous preterm birth (SPTB) at 17–23 (T_1_) and 27–33 (T_2_) weeks of gestation.

		Average of ten five-fold cross validations (cut-off = 0.5)						
		ROC AUC	Sensitivity (%)	Specificity (%)	Positive Predictive Value* (%)	Negative Predictive Value* (%)	False Positive Rate* (%)	False Negative Rate* (%)
**SPTB models with gene expression and significant clinical factors included**								
A	*ZNF605*, *LRRC41*, *PCDHGA12*, *ABT1*, *THBS3*, *VNN1*, history of PTB and history of abortion	0.780	52.4	84.3	61.0	79.2	15.7	47.6
B	*LOC100128908*, *CST13P*, *EEF1D*, *RPH3A*, *TRBV6-6*, *PLEC*, *MIR601*, *ZNF16*, history of abortion and anaemia	0.838	62.3	87.3	67.8	84.5	12.7	37.7
C	*LOC100128908*, *MIR3691*, *LOC101927441*, *CST13P*, *ACAP2*, *ZNF324*, *SH3PXD2B*, *TBX21*, history of abortion and anaemia	0.841	64.7	88.3	70.1	85.4	11.7	35.3
**SPTB models with gene expression only**								
A	*-*	0.703	44.3	81.5	52.5	76.0	18.5	55.7
B	*-*	0.748	46.2	86.5	59.6	79.0	13.5	53.8
C	*-*	0.758	52.6	84.3	58.7	80.7	15.7	47.4

Area under receiver operator curve (ROC AUC)

*As the prevalence of SPTB in this study was 31% (51 SPTB and 114 term deliveries), positive predictive value, negative predictive value, false positive rate and false negative rate must be interpreted with caution as they are dependent on the prevalence of the disease, i.e. PTB in the study population, whilst sensitivity, specificity and ROC AUC are prevalence independent.

**Fig 3:**
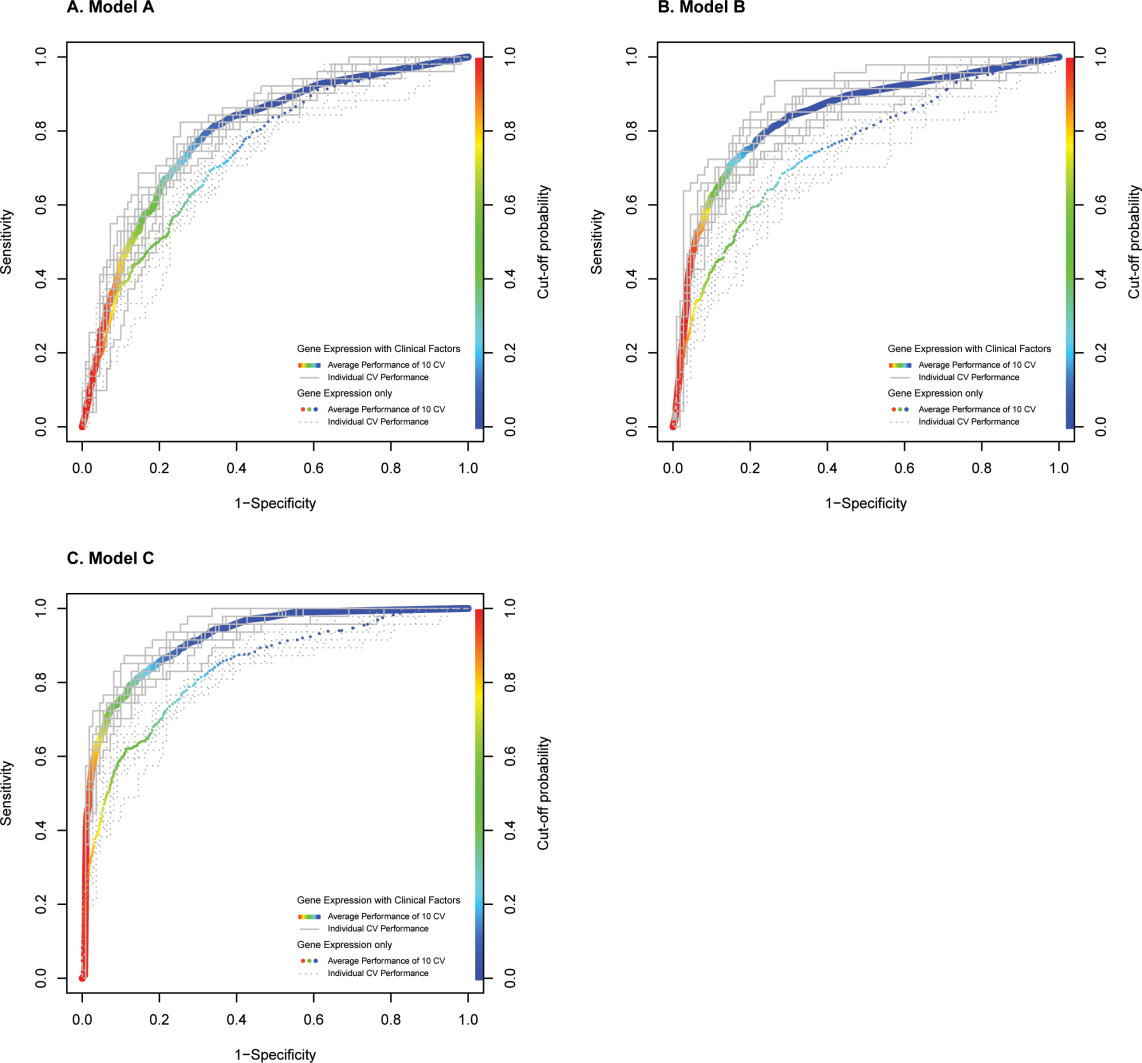
The area under receiver operator characteristic curves of Models A, B and C after ten five-fold cross-validation (CV) runs. These three multivariate models were constructed to identify gene expression associated with spontaneous preterm birth (SPTB) at 17–23 weeks **(A**; Model A) and 27–33 weeks **(B**; Model B); and gene expression fold change from 17–23 to 27–33 weeks of gestation associated with SPTB **(C**; Model C). Models with clinical factors are represented using solid lines; Models without clinical factors are represented using dotted lines. The rainbow bar on the right of each plot displays cut-off probabilities. The colour of the points along the average CV curve reflects its respective cut-off probability to obtain the desired sensitivity and specificity.

The ROC AUCs of Models A, B and C with clinical factors were 11.0%, 12.0% and 10.9% higher than the ROC AUCs of their corresponding Models without clinical factors (Fig 3). This resulted in 18.3%, 34.8% and 23.0% increased sensitivity, and 3.4%, 0.9% and 4.7% increased specificity in Models A, B and C with clinical factors, respectively, when compared to Models without clinical factors. Models B and C were more sensitive than Model A (62.3% and 64.7% versus 52.4%), most likely due to the shorter time frame from sampling at T_2_ to SPTB (average of 4.7 weeks after T_2_).

## Discussion

This study profiled pregnant whole blood mRNA and investigated the association of whole blood gene expression with impending SPTB in asymptomatic women at two clinically relevant time points. T_1_ generally corresponds to when fetal anatomy ultrasound scan is performed and T_2_ is when blood is collected for gestational diabetes screening. This large, paired and unique dataset also provide glimpses of pregnancy progression that result in either SPTB or term delivery. The eleven clinical variables significantly associated with SPTB agree with previous reports [13, 34–39]. Although the association of inflammation with the general physiology of labour at term or preterm gestation is well documented [22, 31, 40, 41], our paired data and gene set enrichment analyses show for the first time, that inflammation is consistently elevated at 17–23 and 27–33 weeks of gestation in the blood of asymptomatic women who had SPTBs compared to women with term deliveries. Lastly, the integration of clinical data alongside gene expression enhanced the sensitivity of our models to predict SPTB.

Gene set analyses provide biological knowledge of how genes interact and orchestrate pathways. Despite not observing any significant gene at FDR<0.05, numerous gene sets were significantly associated with SPTB. We hypothesized that circulating maternal leukocytes pick up ‘signals’ from gestational tissues and respond by altering their gene expression. The most striking gene set enrichment result was that women who had SPTBs have increased interleukin signalling, mainly driven by IL1 and IL6, and leukocyte migration into gestational tissues as early as 18 weeks compared to women who had term deliveries. The early migration of leukocytes into the cervix may accelerate its ripening process and lead to SPTB (Fig 1C) [18, 42]. The increased signalling of IL1 and IL6 can also contribute to SPTB by increasing oxytocin and prostaglandin production leading to accelerated cervical ripening [43–45], early myometrial contractions [46–48] and premature fetal membranes rupture [49, 50].

Our AOB cohort is representative of the pregnant population in urban centres across Canada [23, 51]. The SPTB rate in the state of Alberta is 6.2% [52]. We expected about 110 SPTBs from 1878 AOB participants, but only 51 SPTBs were identified after manual chart review. Thus, our AOB population was not enriched with women at high risk of SPTB. Nevertheless, the predictive models developed in our AOB cohort may offer unique possibilities for research, clinical care and resource utilization. The key to preventing SPTB is the early identification of asymptomatic women at increased risk. The ability to identify these women can aid study groups to focus on high risk women and avoid unnecessary (and expensive) research on those destined for term delivery when evaluating new interventions to prevent PTB. The development of a SPTB predictive tool will also allow further refinement of the subsets of women who will benefit from the existing preventive strategies of progesterone therapy [8, 53], cervical cerclage [54, 55] or pessary [56].

Many research studies have investigated tools to identify high-risk asymptomatic women. For example, the absence of fFN in the cervicovaginal fluid is a classic negative predictor of PTB [14, 15], especially for symptomatic women [57]. Dekker *et al*. reported average predictive capacity for SPTB and PPROM using clinical risk factors, cervical length and uterine artery Doppler ultrasound measurements at 19–21 weeks of gestation [11]. They also reported a minimal overlap of risk factors for SPTB and PPROM, highlighting the heterogeneous condition of PTB. We attempted but were unable to separately assess SPTL and PPROM due to small sample sizes. Kuhrt *et al*. recently developed a validated tool comprising of cervical length, fFN, history of SPTB/PPROM to predict high-risk asymptomatic women with ROC AUCs ranging from 0.77 to 0.99, sensitivity between 54.5% and75.0%, and specificity between 63.5% and 97.7% [13]. The performances of our Models B and C are comparable to Kuhrt *et al*. In addition, it might be more advantageous to screen for biomarkers in maternal blood as blood is easily accessible, minimally invasive and can be collected in most women as part of standard antenatal care [31, 58]. This is in contrast to fFN screening where the test is limited to a subset of eligible women, e.g. had no prior vaginal/cervical examination, unprotected sexual intercourse and/or antepartum haemorrhage.

Models B and C are promising SPTB screening tools since most PTBs occur after 28 weeks of gestation [59]. The slight difference in predictive efficacies between Models B and C, and the simplicity of obtaining one sample at T_2_ makes Model B more clinically applicable. It is important to note that although the predictive efficacies for our Models were reported using a 0.5 cut-off (Table 2), cut-off probability thresholds can be tailored for clinical use, e.g. a higher sensitivity test is required to predict SPTB (Fig 3). Collectively, given the multiple aetiologies of SPTB, a set of diagnostic markers including biochemical, clinical variables, cervical length as well as whole blood gene expression may improve SPTB prediction in asymptomatic women in the future.

In conclusion, this current work has shown that clinical factors and whole blood gene expression are associated with SPTB in asymptomatic women. Gene set enrichment analyses revealed elevated inflammation in women who had SPTBs. Our study did not assess fFN or cervical length data as they were not routinely collected. More studies are needed in other populations to validate our Models and compare them with fFN and/or cervical length. Additional factors such as psychosocial (e.g. prenatal stress and anxiety) can also be included. The ability to implement an effective screening test during antenatal care for SPTB would enable strategic and personalised antenatal care, to improve outcomes for infants and families.

## Supporting Information

S1 Table *limma* outputs for analyses between SPTB and term delivery at (1) T_1_ or (2) T_2_; genes displaying temporal changes between T_1_ and T_2_ in (3) SPTB or (4) term delivery; and (5) genes whose fold change from 17–23 to 27–33 weeks thatwere different between SPTB and term delivery. (XLSX)

S2 Table Significantly enriched gene sets (Gene Ontology Biological Processes, Reactome, KEGG and BioCarta, versions 5.1) using GSEA (FDR<0.05). (XLSX)

S3 Table Microarray and quantitative real time-PCR of 13 unique genes (ranked by fold change). (DOC)

S1 Text Document outlining detailed methodologies. (DOCX)
