# Supplementary material for: Maternal Whole Blood Gene Expression at 18 and 28 Weeks of Gestation Associated with Spontaneous Preterm Birth in Asymptomatic Women
Source: PLoS One. 2016 Jun 22;11(6):e0155191. doi: 10.1371/journal.pone.0155191 (PMC4917227; doi:10.1371/journal.pone.0155191)
Supplement: S3 Table — (DOC) [file pone.0155191.s003.doc]

**S3 Table. Microarray and quantitative real time-PCR of 13 unique** genes (ranked by fold change).

|  | **Forward Primer (5’ to 3’)** | **Reverse Primer (5’ to 3’)** | **Efficiency (%)** | **qRT-PCR** | | **Microarray** | |
| --- | --- | --- | --- | --- | --- | --- | --- |
|  | | | | **Fold Change** | ***p*-value** | **Fold Change** | **FDR** |
| **Spontaneous Preterm Birth: Genes up-regulated at T_2_ compared to T_1_** | | | | | | | |
| *ABCA13* | GCCCTGCTGTGGAAGAATTG | AACAGGATACAAGGCCAGAAGA | 104.0 | 1.95 | <0.001 | 1.49 | <0.001 |
| *MYOF* | CTGGTGGGGAAGTGGAAGATT | CCAAACGTTGGAACAAAGCCT | 104.5 | 1.53 | <0.001 | 1.27 | <0.001 |
| *SASH1* | CTGGAAGTGGAGAAACCCGA | GCTACAGAAGCCAAGCGACT | 98.6 | 1.33 | <0.001 | 1.25 | <0.001 |
| *LAP3* | ACAGGTGCCATGGATGTAGC | CTGTTTCAATGCTGGCCTCG | 92.9 | 1.43 | <0.001 | 1.25 | <0.001 |
| **Spontaneous Preterm Birth: Genes down-regulated at T_2_ compared to T_1_** | | | |  |  |  |  |
| *FCER1A* | CCTGCCATGGAATCCCCTAC | TTCTGAGGGACTGCTAACACG | 98.1 | 0.66 | <0.001 | 0.68 | <0.001 |
| *CPA3* | CCGCTACATCTATGGCCCAAT | CCCAGGTCATAAGCCCAGTC | 96.4 | 0.69 | <0.001 | 0.70 | 0.001 |
| *ABCG1* | TGAGAAAGGACTCCTCGTCCAT | ACCGAGTCCCTCATGATGCT | 90.0 | 0.64 | <0.001 | 0.78 | <0.001 |
| *ABCA1* | AGCACAGGCTTTGACCGATA | GCTCGCAATTACGGGGTTTT | 98.3 | 0.73 | 0.005 | 0.80 | 0.002 |
| **Term Delivery: Genes up-regulated at T_2_ compared to T_1_** | | | |  |  |  |  |
| *OLFM4* | CAGCTGGAGGTGGAGATAAGAA | CCACGATTTCTCGGCGAATG | 94.3 | 2.3 | <0.001 | 1.88 | <0.001 |
| *DEFA3* | CTTGCTGCCATTCTCCTGGT | CATGTTTTTCCTTGAGCCTGGA | 96.1 | 2.7 | <0.001 | 1.86 | <0.001 |
| *DEFA4* | TGCTCTTCAGGTTTCAGGCTC | GCGTGCAGCAGTATGTGAAA | 98.6 | 2.8 | <0.001 | 1.82 | <0.001 |
| *CEACAM8* | TCGTGTCAACCCCAAATTTTTACG | ACAAAGAGTTGTGTTAAAGATGCTG | 92.2 | 2.4 | <0.001 | 1.80 | <0.001 |
| **Term Delivery: Genes down-regulated at T_2_ compared to T_1_** | | | |  |  |  |  |
| *FCER1A* | CCTGCCATGGAATCCCCTAC | TTCTGAGGGACTGCTAACACG | 98.1 | 0.71 | <0.001 | 0.78 | <0.001 |
| *CPA3* | CCGCTACATCTATGGCCCAAT | CCCAGGTCATAAGCCCAGTC | 96.4 | 0.57 | 0.001 | 0.79 | <0.001 |
| *HDC* | GTCAAAGTTGTGGTCGCTGT | TTAGCTCCGCCCTTCAAAGT | 91.0 | 0.83 | 0.124 | 0.82 | <0.001 |
| *ABCG1* | TGAGAAAGGACTCCTCGTCCAT | ACCGAGTCCCTCATGATGCT | 90.0 | 0.81 | <0.001 | 0.83 | <0.001 |
| **Housekeeping Genes** | | |  |  | | | |
| *SDHA* | TGGGAACAAGAGGGCATCTG | CCACCACTGCATCAAATTCATG | 99.5 | - | | | |
| *TBP* | TGCACAGGAGCCAAGAGTGAA | CACATCACAGCTCCCCACCA | 95.8 | - | | | |
| *YWHAZ* | ACTTTTGGTACATTGTGGCTTCAA | CCGCCAGGACAAACCAGTAT | 91.0 | - | | | |
