# Supplementary material for: Maternal Whole Blood Gene Expression at 18 and 28 Weeks of Gestation Associated with Spontaneous Preterm Birth in Asymptomatic Women
Source: PLoS One. 2016 Jun 22;11(6):e0155191. doi: 10.1371/journal.pone.0155191 (PMC4917227; doi:10.1371/journal.pone.0155191)
Supplement: S1 Text — (DOCX) [file pone.0155191.s004.docx]

**S1 Text: Supplementary Methods**

- 1. **DIFFERENTIAL GENE EXPRESSION ANALYSES**

To compare between spontaneous preterm labour (SPTL) and preterm prelabour rupture of membranes (PPROM) at time point 1 (T1, 17-23 weeks) and at time point 2 (T2, 27-33 weeks)

- Gestational age, pre-pregnancy BMI, polyhydramnios and APH after 20 weeks were adjusted for in the limma analyses. No differentially expressed genes were obtained hence SPTL and PPROM were combined into a spontaneous preterm birth (SPTB) group.

To compare between SPTB and Term delivery at T1

- Gestational age, alcohol consumption, history of PTB, history of abortion and urinary tract infection (UTI) present before T1 were adjusted for in the limma analysis.

To compare between SPTB and Term delivery at T2

- Gestational age, alcohol consumption, history of PTB, history of abortion, UTI present before T2 and anaemia present before T2 were adjusted for in the limma analysis

To compare temporal gene expression between T2 and T1 in SPTB

- Women were accounted for in this paired limma analysis.

To compare temporal gene expression between T2 and T1 in Term

- Women were accounted for in this paired limma analysis.

To compare gene expression fold change differences between SPTB and Term

- Women were accounted for in this paired limma analysis

**1.2 CONSTRUCTING MULTIVARIATE MODELS ASSOCIATED WITH SPONTANEOUS PRETERM BIRTH**

1. Model A
2. Performed limma and adjusted for gestational age at T1. Selected genes whose |t value|>3 (*n*=320).
3. Performed univariate analysis (logistic regression) on each selected gene from A. by adjusting for history of preterm birth and history of abortion (multivariate analyses’ significant clinical factors before T1). Chose top 20 genes for C (to avoid overfitting).
4. Performed multivariate logistic regression (stepwise selection) using genes from B., and history of preterm birth, history of abortion and gestational age at T1 were fixed in the model.
5. Model B
6. Performed limma and adjusted for gestational age at T2. Selected genes whose |t value|>3 (*n*=195).
7. Performed univariate analysis (logistic regression) on each selected gene from A. by adjusting for history of abortion and anaemia (multivariate analyses’ significant clinical factors before T2). Chose top 20 genes for C (to avoid overfitting).
8. Performed multivariate logistic regression (stepwise selection) using genes from B., and history of abortion and anaemia and gestational age at T2 were fixed in the model.
9. Model C
10. Performed limma and adjusted for gestational age at T1 and the number of weeks between T1 and T2. Extracted genes whose |t value|>3 (*n*=115).
11. Performed univariate analysis (logistic regression) on each selected gene from A. by adjusting for history of abortion and anaemia (multivariate analyses’ significant clinical factors before T2), and gestational age at T1. Chose top 20 genes for C (to avoid overfitting).
12. Performed multivariate logistic regression (stepwise selection) using genes from B., and history of abortion, anaemia and gestational age at T1 were fixed in the model.

Gestational age at T1 is used (instead of T2) in C as a way to “adjust” for the temporal gene expression obtained in A.

**1.3 FIVE-FOLD CROSS-VALIDATION OF MULTIVARIATE MODELS WITH AND WITHOUT CLINICAL FACTORS**

1. Each Model was subjected to ten five-fold cross-validation with gene selection occurring at every fold, with clinical factors and gestational age fixed in the model. The selected genes at each fold were recorded.
2. To evaluate the importance and effect of adjusting gene expression with clinical factors, models were built using gene expression only. This was carried out by immediately repeating the training of the exact fold in 1. using the recorded genes and excluding the clinical factors.
